# Draft Genome Sequence of a *Delftia* sp., a Member of an Electroactive Community Enriched from Wastewater from the Indian Institute of Technology Delhi, India

**DOI:** 10.1128/mra.01121-22

**Published:** 2023-03-15

**Authors:** Nirmal Singh Mahar, Kartik Aiyer, Juwayria, Lucinda Elizabeth Doyle, Ishaan Gupta

**Affiliations:** a Department of Biochemical Engineering and Biotechnology, Indian Institute of Technology Delhi, New Delhi, India; b Center for Electromicrobiology, Aarhus University, Aarhus, Denmark; Wellesley College

## Abstract

The draft genome sequence of *Delftia* sp. is reported here. The genome was recovered from a mixed-species electroactive community in a microbial fuel cell that had been inoculated with wastewater from the Indian Institute of Technology Delhi, India. Sequencing was performed using Nanopore technology.

## ANNOUNCEMENT

Certain *Delftia* species are members of an electroactive community participating in extracellular electron transfer to the electrode in bioelectrochemical systems, with electron transfer proposed to occur via soluble electron shuttles (1). Since they are commonly found in wastewaters, *Delftia* species have potentially important roles in wastewater treatment plants and anaerobic digesters for waste remediation and energy recovery.

*Delftia* sp. was enriched in dual-chambered microbial fuel cells inoculated with domestic wastewater from the Indian Institute of Technology (IIT) Delhi. The culture was derived by inoculating a growth medium with wastewater (10% [vol/vol]) collected from the IIT Delhi campus. The nutrient medium was adapted from the report by Bretschger et al. (2) and consisted of the following: CH_3_COONa, 1.00 g/L; NH_4_Cl, 0.31 g/L; NaH_2_PO_4_·H_2_O, 5.38 g/L; Na_2_HPO_4_, 8.66 g/L; KCl, 0.13 g/L; trace mineral solution, 12.5 mL/L; amino acid solution, 12.5 mL/L. The trace mineral solution consisted of the following: C_6_H_9_NO_3_, 11.24 mg/L; MgSO_4_·7H_2_O, 14.65 mg/L; MnSO_4_·H_2_O, 4.99 mg/L; NaCl, 10 mg/L; FeSO_4_·7H_2_O, 564 μg/L; CaCl_2_·2H_2_O, 1 mg/L; CoCl_2_·6H_2_O, 545 μg/L; ZnCl_2_, 1.3 mg/L; CuSO_4_·5H_2_O, 100 μg/L; KAl(SO_4_)_2_·12H_2_O, 100 μg/L; H_3_BO_3_, 100 μg/L; Na_2_MoO_4_·2H_2_O, 212 μg/L; Na_2_WO_4_·2H_2_O, 250 μg/L. The amino acid solution consisted of 20 mg/L l-glutamic acid, l-arginine, and l-serine. The final pH of the medium was adjusted to 7. The microbial fuel cells were operated at room temperature throughout the duration of the experiment. After inoculation in microbial fuel cells, the culture was actively monitored for voltage generation over a period of 28 days, after which DNA was extracted from the electroactive community for sequencing.

DNA was extracted from the enriched community using a ZymoBIOMICS DNA miniprep kit (D4300; Zymo Research, Irvine, CA, USA) according to the manufacturer’s instructions. The draft genome sequence was determined by Nanopore sequencing on a MinION Mk1B sequencer with R9.4.1 chemistry and library protocol SQK-LSK110 (Oxford Nanopore Technologies, Oxford, UK). A total of 2.11 million reads were obtained, with an average read length of 2.9 kbp. Of these 2.11 million reads, 61,616 reads belonged to the *Delftia* genus. The adapter sequences were initially trimmed from the raw reads with Porechop version 0.2.4 (3) and assembled into contigs with Flye version 2.9 (4) with the metagenome option enabled. The draft assembly, consisting of 1,961 contigs, was further refined using three rounds with Racon version 1.4.3 (5) and one round with Medaka version 1.5.0 (https://github.com/nanoporetech/medaka). Since the sample had a metagenomic origin, the taxonomic classification was performed using kraken2 version 2.1.2 (6), and contigs were binned into genus-level bins using the python script extract_kraken_reads.py provided by KrakenTools version 1.2 (7). BBTools (8) was used to filter contigs with lengths of >500 bp, and BLASTN version 2.12.0+ (9) with the nucleotide collection database was used for species-level identification.

The bin for the *Delftia* genus contained 105 contigs, and contigs that belonged to the *Delftia* genus were scaffolded using Ragtag version 2.1.0 (10). An assembly with a length of 7,751,118 bp, with 74 contigs (GC content, 65.88%), was obtained. The assembly metrics were generated using Quast version 5.0.2 (11), with an *N*_50_ value of 6,847,017 bp and 39.99 Ns per 100 kbp.

To assess the completeness of the genome, Benchmarking Universal Single-Copy Orthologs (BUSCO) version 5.3.0 (12) with the bacteria_odb 10 database was used. Of the 125 BUSCOs generated, 122 were complete (98.4%), 2 were fragmented (shorter than expected), and 1 was missing. Of the 122 complete BUSCOs, 121 were single copy and 1 was duplicated. Finally, the genome was annotated using the NCBI Prokaryotic Genome Annotation Pipeline (PGAP) version 2022-04-14.build 6021 (13). The annotation resulted in 6,952 predicted coding sequences (CDSs), 9 rRNAs, 86 tRNAs, 3 noncoding RNAs, 3 CRISPR arrays, and 417 pseudogenes.

**FIG 1:**
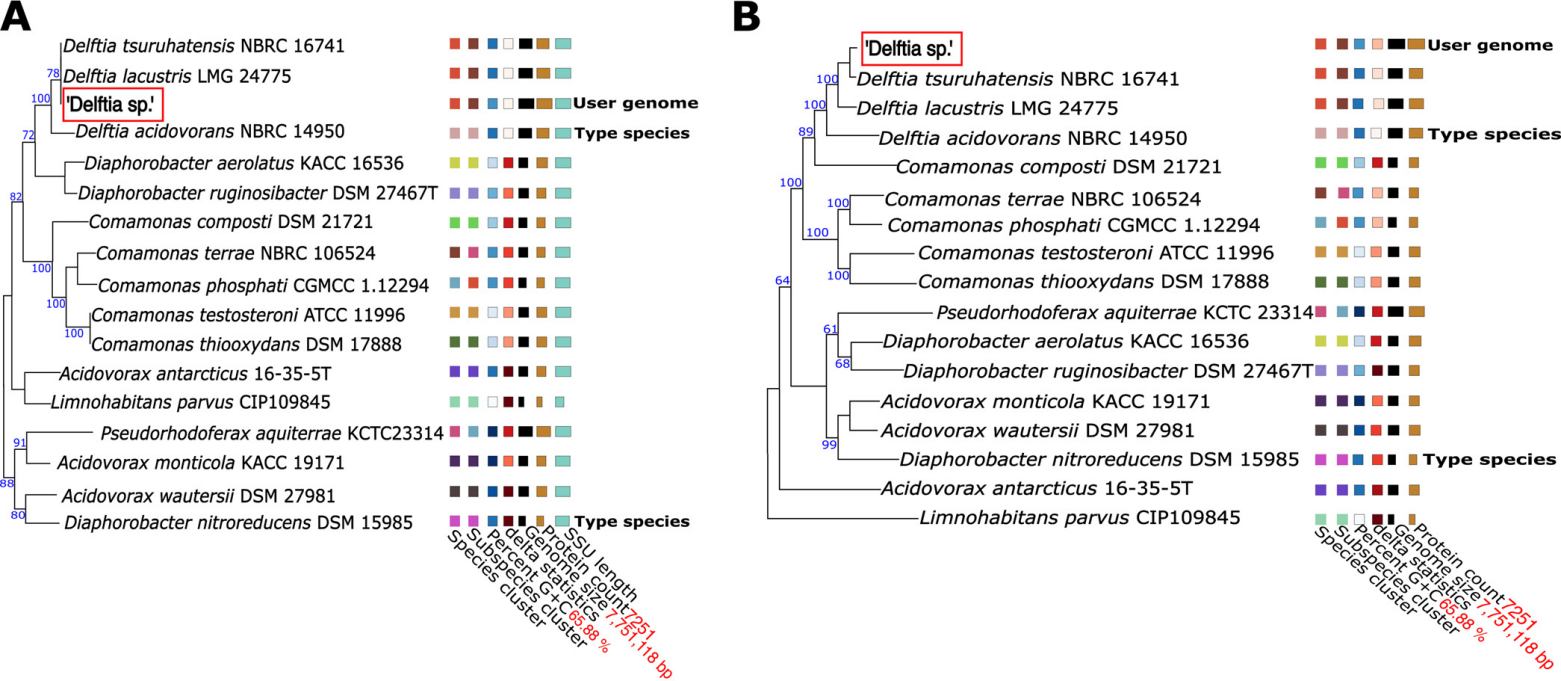
(A) Phylogenetic tree based on 16S rRNA gene sequences, generated using TYGS. (B) Phylogenetic tree based on whole-genome sequences, generated using TYGS, which confirms the genus as *Delftia.*

For further analysis, the Type (Strain) Genome Server (TYGS) was used for comparison of the 16S rRNA gene (14). The 16S rRNA gene sequence of the *Delftia* sp. belonged to the same clade of 16S rRNA gene sequences as did those of Delftia tsuruhatensis NBRC 16741 and Delftia lacustris LMG 24775 (Fig. 1A). In addition, the 16S rRNA gene sequence of the *Delftia* sp. was extracted from the final assembly using barrnap version 0.9 (https://github.com/tseemann/barrnap). This sequence showed 99.3% sequence similarity with those of Delftia tsuruhatensis NBRC 16471 (GenBank accesion number GCA001571325.1) and Delftia lacustris strain 332 (GenBank accession number GCA900107225.1) when aligned using BLASTn version 2.12.0+ with the 16S rRNA sequence database.

### Data availability.

This whole-genome shotgun project has been deposited in DDBJ/ENA/GenBank under the accession number JANFAT000000000 and the BioProject accession number PRJNA824721. The version described in this paper is version JANFAT010000000. Raw reads are accessible via NCBI with the SRA accession number SRR22212390.
